# Faculty members' earthquake preparedness levels and their related factors: a cross-sectional study from a university in a high-risk earthquake zone in Turkey

**DOI:** 10.5249/jivr.vo113i2.1513

**Published:** 2021-07

**Authors:** Raziye Ozdemir, Cigdem Demir, Binali Catak

**Affiliations:** ^*a*^ Department of Occupational Health and Safety, Faculty of Health Sciences, Karabuk University, 78050 Karabuk, Turkey.; ^*b*^ Department of Public Health, Health Sciences Institute, Karabuk University, Karabuk, Turkey.; ^*c*^ Department of Public Health, Faculty of Medicine, Kafkas University, Kars, Turkey.

**Keywords:** Earthquake preparedness, Risk reduction, Safety measures, The attitude of faculty members, Turkey

## Abstract

**Background:**

This cross-sectional study aimed to determine the earthquake preparedness levels and related factors of the faculty members working at a university located in a province in a first-degree earthquake zone in Turkey.

**Methods:**

The total number of faculty members at the university is 457, including 314 assistant professors, 63 associate professors, and 80 full professors. The study group included 177 (38.7%) of 457 faculty members. The dependent variable of the study is the attitudes of the faculty members towards earthquake preparedness. The independent variables were age, gender, marital sta-tus, having children, living with or without children, duration of residency in Karabuk. In addition, these were also included as variables, respectively, type of housing, property ownership, work experience, exposure to natural disasters in the past, and the awareness that Karabuk is a first-degree risk earthquake zone. The data were collected using a descriptive questionnaire and Mulilis-Lippa Earthquake Preparedness Scale. Data were analyzed using chi-square tests and binary logistic regression model and SPSS 20.0 software.

**Results:**

There was only one (0.6%) faculty member who stated that he meets all of the preparatory criteria of the Mulilis-Lippa Earthquake Preparedness Scale. The probability of low materials preparedness score increased by 2.31 (95% CI 1.1-4.7) and 4.53 (95% CI 1.4-14.4) when the faculty members were renters and working as faculty members for 15 years and over, respectively. Being a renter also increased the probability of common knowledge and skill score by 1.95 (95% CI 1.0-3.8).

**Conclusions:**

This study showed that earthquake preparedness levels among the faculty members are insuffi-cient and suggests that motivation of the faculty members’ motivation to be appropriately pre-pared for future earthquake case should be increased.

## Introduction

An earthquake is a natural event that occurs suddenly and unexpectedly. The country’s infrastructure, population, economy, and social structure can be seriously damaged, especially if the earthquake is severe and the preparedness measures for the earthquake are insufficient. In recent years, the effects of disasters, including earthquakes, are felt more intensely than in the past due to features such as increasing population density, industrialization, and urbanization.^1^

More than a million earthquakes occur globally; as a consequence, more than one million people have lost their lives due to earthquake incursions only in the last 20 years.^1^ Nearly 90% of the earthquake fatalities occur in developing countries. More than 80% of the deaths caused by earthquakes in the last century have appeared in nine countries, including Turkey, Armenia, Chile, China, Guatemala, Iran, Italy, Japan, and Peru.^1^ Moreover, many people are injured because of earthquakes and ended up homeless.^2^ The lack of awareness and preparedness, poor engineering design and construction practices, and corrupt practices in the construction sector in developing countries are considered serious drawbacks.^2,3^ Located in the Alpine-Himalayan orogenic belt, Turkey is one of the world’s most active regions concerning earthquakes. The vast majority (98%) of the population lives under an earthquake risk in Turkey. Additionally, 98% of the leading industrial establishments are in earthquake zones, while 73% are located at active fault zones. Furthermore, 95% of dams are in active earthquake zones.^4^ Two hundred and twenty-three major earthquakes occurred between 1900 and 2009 in Turkey during which, 86 thousand people have lost their lives, while the authorities have identified destroyed or profoundly damaged housing affecting approximately 549 thousand people.^5^ On August 17, 1999, a 7.6 on the Richter Scale earthquake had struck the Marmara region of Turkey, resulting in 17,118 human deaths.

Earthquake preparedness is crucial for the elimination of the damages attributed to earthquakes. Taking personal precautions allows human lives sustaining by reducing death and injuries that may arise after an intense earthquake episode.^6^ For instance, fires related injuries caused by broken gas lines can be prevented by learning how to close a gas line. Similarly, securing heavy furnishings such as a bookcase to the walls prevents extensive injuries. Materials such as first aid supplies, canned food, and bottled water can increase the chances of survival after an earthquake. ^7^ At an individual or institutional level, there are severe deficiencies in Turkey about taking measures against disasters, reducing the potential damages, and planning for readiness to disasters.^8-11^ In a study conducted in Antalya by Dedeoglu, it was reported that, among 198 people, only 1 (0.5%) has an earthquake home emergency kit and only 21% of the participants have received first aid training. Furthermore, over half of the participants (58%) adopted an attitude against surviving an earthquake which is ones’ fate is sealed.^8^ Similarly, in other developing countries, the level of preparation of individuals is low. A study involving 2,686 people in China revealed that less than 5% of the participants were classified decently prepared,^12^ while in the USA, the level of disaster preparedness of individuals is higher. In a study conducted by the US Federal Emergency Management Agency (FEMA), it was reported that 57% and 45% of the individuals have an emergency kit in their home and workplace, respectively, whereas 34% of them own the related equipment in their car.^13^ Attitudes and behaviors of individuals to disaster preparedness may vary depending on the region and the characteristics of the community in which they live. Studies indicate that factors such as age,^14,15^ gender,^11,12^ education level,^8,14,16^ property ownership,^14,15^ previous exposure to a disaster,^12,17,18^ household income level,^12,14,15,18^ occupation,^18^ confidence in government efforts, number of dependents in a household,^17^ social assurance,^19^ higher levels of knowledge about emergency, positive attitudes towards emergency preparedness,^12^ race, type of housing^15^ are associated with the disaster preparedness attitudes and behaviors of individuals. 

This study aims to determine the earthquake preparedness of faculty members who work in a university located in the first-degree earthquake zone in Turkey and the associated factors.

## Materials and Methods

The cross-sectional study was conducted at Karabuk University. A total of 457 faculty members are employed at the university. According to their profession, there are 314 assistant professors, 50 associate professors, and 80 full professors. Therefore, the sample size was determined as 209 with a % 50 prevalence, 5% margin of error, and 95% confidence interval using Epiinfo Statcalc 7.2. Thirty-two faculty members refused to participate in the study. Thus, the final study group comprised 177 (84.7%) faculty members in total. 

The dependent variable is the level of earthquake preparedness. Independent variables were age, gender, marital status, having children, living with or without children, duration of residency in Karabuk, type of housing, property ownership, work experience, awareness of Karabuk’s being in a first-degree seismic zone, and exposure to a natural disaster in the past. 

The data were collected from the faculty members through face-to-face interviews from March to June 2018. Two forms were used as data collection tools. The first one was a form that included 12 questions and questioned the descriptive characteristics of faculty members, while the second one was the Revised and Translated Mullis-Lippa Earthquake Preparedness Scale (MLEPS), which includes 28 items. The MLEPS developed by Mulilis and Lippa aims to measure individuals’ earthquake preparedness behaviors and the perceived difficulty of becoming prepared for earthquakes.^20^ The MLEPS has been translated and adapted into Turkish by Sakiroglu in 2005. In the Turkish version, a section was added to measure the perceived usefulness of each preparation item (Cronbach’s alpha is 0.78 for the earthquake preparedness behaviors, 0.86 for the perceived difficulty, 0.80 for the perceived usefulness).^21^ In this study, Cronbach’s alpha was found 0.75 for the earthquake preparedness behaviors, 0.89 for the perceived difficulty, and 0.94 for the perceived usefulness.

The MLEPS questions the earthquake preparedness behaviors of the individuals in three categories: 1) material preparedness, 2) planning, and 3) knowledge and skill. Material preparedness contains the relevant precautions like keeping the materials and equipment at home continuously, which may be helpful in case of a disaster, such as food, water, fire extinguisher, first aid kit, and raising the household’s awareness about fixing the tall furniture to the walls, and familiarity with the electricity, gas and water networks. Planning activities determine a safe place where the household can meet outside or inside the house. Knowledge and skill refer to the practices of individuals to participate in meetings to establish disaster preparedness, obtain first aid training, and improve knowledge and skills by reading written material. Individuals are asked to report whether they are prepared, unprepared, or unsure concerning the preparedness behavior described in each item. Individuals also can choose one of very much, a little, and not at all responses for the degree of difficulty and usefulness of fulfilling each preparatory criterion. 

The data were summarized as percentages. A detailed analysis was made with the findings from the part of the earthquake preparedness behaviors of the individuals of the scale. The answers given to the MLEPS earthquake preparedness questions were rated as prepared= 1, unprepared, and unsure= 0 to assess the relationship between dependent and independent variables. The points that the study group received from three preparedness categories of the scale were classified as low (< median value) and high (≥ median value) by taking the breakpoints of the median values. The chi-squared test was used to assess the factors associated with the earthquake preparedness behaviors of faculty members. The variables with a value of p<0.05 in the chi-squared test analysis were included in the binary logistic regression model. The variables in the binary logistic regression analysis were considered statistically significant at p<0.05. The data analysis was performed using SPSS 20.0 software.

## Results

Table 1 shows the descriptive characteristics of the study population. The majority (74.4%) of faculty members were in the age between 27 and 46 years. Males comprised 78.0% of the study population. Most faculty members (85.3%) were married, and 75.1% had at least one child. Less than a half (44.6%) of the faculty members were homeowners. Almost one-fifth (24.3%) of the faculty members reported that they had experienced a natural disaster in the past. The level of those who knew that Karabuk is a first-degree earthquake zone was 37.9% (Table 1).

**Table 1:** The descriptive characteristics of the faculty members

Variables	n	%
Age*		
27-46	128	74.4
47-66	44	25.6
**Gender**		
Female	39	22.0
Male	138	78.0
**Marital status**		
Single	26	14.7
Married	151	85.3
**Having children**		
Have a child	133	75.1
Don’t have a child	44	24.9
**Living with child/children**		
Yes	126	71.2
No	7	4.0
Don’t have a child	44	24.9
**Duration of residency in Karabuk **		
≤5 years	88	49.7
≥6 years	89	50.3
**Type of housing**		
Apartment	170	96.0
House	7	4.0
**Property ownership**		
Homeowner	79	44.6
Renter	98	55.4
**Work experience as a faculty member**		
≤15 years	133	75.1
≥16 years	44	24.9
**Awareness of Karabuk’s presence in a first-degree earthquake zone **		
Aware	67	37.9
Unaware	110	62.1
**Exposure to a natural disaster in the past**		
Yes	43	24.3
No	134	75.7
**Total**	**177**	**100.0**

*Five (2.8%) faculty members who did not indicate their age were excluded.

Table 2 describes the earthquake preparedness behaviors of the faculty members and their perception of difficulty and usefulness of earthquake preparation criteria. The responses to the earthquake preparedness questions are presented under three categories: 1) material preparedness, 2) planning and 3) knowledge and skill. Only one (0.6%) faculty member claimed that he fulfilled all the preparation criteria.

**Table 2:** The earthquake preparedness behaviors of faculty members and their perception of difficulty and usefulness of each preparation items (n= 177)

The earthquake preparedness category of the MLEPS	Preparedness behavior		Difficulty of preparedness		Usefulness of preparedness	
	Prepared	Unprepared/unsure	Not at all	A little/very much	Not at all	A little/very much
	n (%)*	n (%)*	n (%)*	n (%)*	n (%)*	n (%)*
**1. Materials preparedness**						
**a) Material supply **						
An operating flashlight	81 (45.8)	96 (54.2)	150 (84.7)	27 (15.3)	5 (2.8)	172 (97.2)
Extra batteries for the flashlight	41 (23.3)	135 (76.7)	146 (82.5)	31 (17.5)	7 (4.0)	170 (96.0)
An operating transistor radio	19 (10.7)	158 (89.3)	129 (72.9)	48 (27.1)	11 (6.2)	166 (93.8)
Extra batteries for the transistor radio	20 (11.4)	155 (88.6)	128 (72.3)	49 (27.7)	11 (6.2)	166 (93.8)
A complete first-aid kit	51 (29.0)	125 (71.0)	122 (68.9)	55 (31.1)	2 (1.1)	175 (98.9)
Bottled water (four gallons)	60 (34.3)	115 (65.7)	137 (77.4)	40 (22.6)	4 (2.3)	173 (97.7)
At least four days’ supply of canned food	35 (19.9)	141 (80.1)	113 (63.8)	64 (36.2)	5 (2.8)	172 (97.2)
An operating fire extinguisher	32 (18.3)	143 (81.7)	107 (60.5)	70 (39.5)	6 (3.4)	171 (96.6)
Emergency telephone number list	40 (23.0)	134 (77.0)	145 (81.9)	32 (18.1)	9 (5.1)	168 (94.9)
**b) Utilities **						
Do you know the location of the following utilities?						
Water valve	165 (93.2)	12 (6.8)	165 (93.2)	12 (6.8)	3 (1.7)	174 (98.3)
Gas valve	167 (94.4)	10 (5.6)	167 (94.4)	10 (5.6)	1 (0.6)	176 (99.4)
Electric power switch	173 (97.7)	4 (2.3)	168 (94.9)	9 (5.1)	1 (0.6)	176 (99.4)
Do you know how to shut down the following utilities?						
Water valve	167 (94.4)	10 (5.6)	163 (92.1)	14 (7.9)	5 (2.8)	172 (97.2)
Gas valve	165 (93.2)	12 (6.8)	166 (92.1)	14 (7.9)	5 (2.8)	172 (97.2)
Electric power switch	171 (96.6)	6 (3.4)	164 (92.7)	13 (7.3)	4 (2.3)	173 (97.7)
**c) Fixation **						
Water heaters/combi boilers	139 (79.0)	37 (21.0)	103 (58.2)	74 (41.8)	7 (4.0)	170 (96.0)
Cupboards	51 (28.8)	126 (71.2)	68 (38.4)	109 61.6)	2 (1.1)	175 (98.9)
Tall furniture	44 (24.9)	133 (75.1)	63 (35.6)	114 (64.4)	2 (1.1)	175 (98.9)
Heavy objects placed high on walls	67 (38.3)	108 (61.7)	88 (49.7)	89 (50.3)	4 (2.3)	173 (97.7)
**2. Planning**						
Does your household have a meeting place to come together after a possible earthquake?	16 (9.0)	161 (91.0)	142 (80.2)	33 (19.8)	6 (3.4)	171 (96.6)
During a possible earthquake, does your household have a plan for a safe place?	40 (22.9)	135 (77.1)	139 (78.5)	38 (21.5)	7 (4.0)	170 (96.0)
**3. Knowledge and skill**						
Do you know the location of a health center in your neighborhood?	169 (95.5)	8 (4.5)	164 (92.7)	13 (7.3)	1 (0.6)	176 (99.4)
Do you read material on earthquake preparedness?	108 (61.4)	68 (38.6)	137 (77.4)	40 (22.6)	2 (1.1)	175 (98.9)
Do you attentively listen to or watch news/messages about earthquake preparedness on media?	117 (66.1)	60 (33.9)	133 (75.1)	44 (24.9)	5 (2.8)	172 (97.2)
Do you attend meetings for establishing earthquake preparedness?	36 (20.3)	141 (79.7)	82 (46.3)	95 (53.7)	4 (2.3)	173 (97.7)
Have you attended a first aid course?	84 (47.7)	92 (52.3)	101 (57.1)	76 (42.9)	2 (1.1)	175 (98.9)
Does your household have earthquake insurance?	142 (80.2)	35 (19.8)	123 (69.5)	54 (30.5)	11 (6.2)	166 (93.8)
Have the officials made the control of resistance of your house?	52 (29.4)	125 (70.6)	78 (44.1)	99 (55.9)	6 (3.4)	171 (96.6)

*Row percentage

**1) Material Preparedness**

a) Material supply: According to the faculty members’ statements, 45.8% of them had been equipped with an operating flashlight; 29.0% of them had a complete first-aid kit; 23.0% of them were aware of an emergency telephone number list; 19.9% of them had stored canned food; 34.3% of them prepared bottled water; 10.7% of them owned an operating transistor radio, and 18.3% of them prepared an operating fire extinguisher in case of an emergency. There was only one (0.6%) faculty member who fulfilled all the preparations for material supply, while 115 (65.0%) of them prepared at least one, and 61 (34.5%) members did not have any of them. The percentage of the faculty members who believe that supply is not difficult is: a) 84.7% for flashlight, b) 72.9% for radio, c) 68.9% for first aid kit, d) 77.4% for bottled water, e) 63.8% for canned food, f) 60.5% for a fire extinguisher, and g) 81.9% for emergency telephone numbers list. The majority of the faculty members reported that it is helpful to prepare these materials (Table 2).

b) Precautions for the electric switch, gas, and water valves: Ninety-three percent (93%) of the faculty members know the place of the water valve in their house, while 94.4% of them is aware of the gas/natural gas valve and 97.7% of them knows the electrical power switch. Furthermore, 95.4% of the faculty members know how to turn off the water valve, 94.3% of them know how to turn off the gas/natural gas valve, and 97.7% of them how to turn off the electric power. More than 90% of the faculty members claimed that it is not difficult to fulfill these preparatory measures, and almost all of them stated that these preliminary measures practical Table 2).

c) Fixation: Seventy-nine percent (79%) of the faculty members reported that they fixed their water heaters or combi boilers, 38.3% of them secured the heavy objects, setting them to the walls, 28.8% of them fixed the cupboards, and 24.9% of them fastened the tall furniture to the walls. While 25 (14.1%) of the faculty members reported that they fixed all of the heavy objects in their home, 31 (17.5%) of them had not secured any entity. The percentage of faculty members who think that fixation is complex was higher compared to other preparatory measures. However, almost all of them reported that these measures are useful (Table 2).

**2) Planning**

Considering preventive measure plans, 16 (9.0%) faculty members have determined a meeting place with their family members. In comparison, 40 (22.9%) faculty members have identified a safe place to shelter during the earthquake the home after an earthquake occurs. Approximately 80% of the faculty members believe that these measures are not complicated, and 96% of them believe these are useful to apply for (Table 2).

**3) Knowledge and Skills**

Sixty-one percent (61.4%) of the faculty members reported that they read tutorials about earthquake preparedness, 47.7% of them have received first aid training, 80.2% of them have earthquake insurance, and 29.4% of them stated that the experts checked the control of the earthquake resistance of their houses. In this category, the most difficult precautionary measure for the faculty members was to have the officials check the resistance of their homes (Table 2).

The percentage of scores that was lower than the median value (9) for the material preparedness category of the MLEPS was higher among younger faculty members compared to older ones (p=0.034), singles than married ones (p=0.034), renters than homeowners (p=0.002), as well as faculty members with ≤15 years of work experience compared to ones with ≥16 years (p=0.001) (Table 3).

**Table 3:** The factors associated with the earthquake preparedness behaviors of the faculty members by three categories of MLEPS

	Materials preparedness score				Planning score				Knowledge and skill score			
	Low	High			Low	High			Low	High		
	n (%)*	n (%)*	X^2**^	p	n (%)*	n (%)*	X^2**^	p	n (%)*	n (%)*	X^2**^	p
**Age**												
27-46	55 (43.0)	73 (57.0)	4.471	0.034	100 (78.1)	28 (21.9)	2.606	0.106	51(39.8)	77 (60.2)	0.897	0.344
47-66	11 (25.0)	33 (75.0)			29 (65.9)	15 (34.1)			14 (31.8)	30 (68.2)		
**Gender**												
Female	18 (46.2)	21 (53.8)	1.081	0.298	29 (74.4)	10 (25.6)	0.049	0.824	12 (30.8)	27 (69.2)	0.909	0.340
Male	51 (37.0)	87 (63.0)			105 (76.1)	33 (23.9)			54 (39.1)	84 (60.9)		
**Marital status**												
Single	15 (57.7)	11 (42.3)	4.485	0.034	20 (76.9)	6 (23.1)	0.025	0.876	14 (53.8)	12 (46.2)	3.573	0.059
Married	54 (35.8)	97 (64.2)			114 (75.5)	37 (24.5)			52 (34.4)	99 (65.6)		
**Having children**												
Have a child	47 (35.3)	86 (64.7)	2.988	0.084	101 (75.9)	32 (24.1)	0.016	0.900	42 (31.6)	91 (68.4)	7.458	0.006
Don’t have a child	22 (50.0)	22 (50.0)			33 (75.0)	11 (25.0)			24 (54.5)	20 (45.5)		
**Living with child/children (n= 133)**												
Yes	45 (35.7)	81 (64.3)	0.148	0.700	97 (77.0)	29 (23.0)	1.429	0.232	38 (30.2)	88 (69.8)	2.235	0.135
No	2 (28.6)	5 (71.4)			4 (57.1)	3 (42.9)			4 (57.1)	3 (42.9)		
**Duration of residency in Karabuk**												
≤5 years	37 (42.2)	51 (58.0)	0.690	0.406	70 (79.5)	18 (20.5)	1.403	0.236	37 (42.0)	51 (58.0)	1.694	0.193
≥6 years	32 (36.0)	57 (64.0)			64 (71.9)	25 (28.1)			29 (32.6)	60 (67.4)		
**Type of housing**												
Apartment	66 (38.8)	104 (61.2)	0.046	0.830	129 (75.9)	41 (24.1)	0.073	0.788	64 (37.6)	106 (62.4)	0.237	0.627
House	3 (42.9)	4 (57.1)			5 (71.4)	2 (28.6)			2 (28.6)	5 (71.4)		
**Property ownership**												
Homeowner	21 (26.6)	58 (73.4)	9.582	0.002	57 (72.2)	22 (27.6)	0.906	0.341	21 (26.6)	58 (73.4)	6.592	0.010
Renter	48 (49.5)	49 (50.5)			76 (78.4)	21 (21.6)			44 (45.4)	53 (54.6)		
**Work experience as a faculty member**												
≤15 years	61 (45.9)	72 (54.1)	10.652	0.001	103 (77.4)	30 (22.6)	0.878	0.349	54 (40.6)	79 (59.4)	2.512	0.113
≥16 years	8 (18.2)	36 (81.8)			31 (70.5)	13 (29.5)			12 (27.3)	32 (72.7)		
**Awareness of Karabuk’s presence in a first-degree earthquake zone**												
Aware	24 (35.8)	43 (64.2)	0.453	0.501	24 (35.8)	43 (64.2)	0.453	0.501	19 (28.4)	48 (71.6)	3.676	0.055
Unaware	45 (40.9)	65 (59.1)			45 (40.9)	65 (59.1)			47 (42.7)	63 (57.3)		
**Exposure to a natural disaster in the past**												
Yes	17 (39.5)	26 (60.2)	0.007	0.932	28 (65.1)	15 (34.9)	3.463	0.063	11 (25.6)	32 (74.4)	3.329	0.068
No	52 (38.8)	82 (61.2)			106 (79.1)	28 (20.9)			55 (41.0)	79 (59.0)		
**Total**	69 (39.0)	108 (61.0)			134 (75.7)	43 (24.3)			66 (37.3)	111 (62.7)		

*Row percentage **Chi-square test

No significant relationship was found between the planning category scores of the faculty members and the independent variables (p>0.05) (Table 3).

The median value for the knowledge and skill scores was found 4. The knowledge and skill score was lower for the renters compared to the homeowners (p=0.010). In addition, the score of this category was lower for the faculty members who had no children compared to those who had children (p= 0.006) (Table 3).

Table 4 presents the binary logistic regression analysis with the results that were significant in the chi-square test. The probability of low materials preparedness score increased by 2.31 (95% CI 1.1-4.7) and 4.53 (95% CI 1.4-14.4) times when the faculty members were renters and working as faculty members for ≤15 years, respectively. Being a renter also increased the probability of common knowledge and skill score by 1.95 (95% CI 1.0-3.8) times (Table 4).

**Table 4:** Logistic regression analysis on earthquake preparedness

Variable	Materials preparedness			Knowledge and skill		
	OR	p	95% CI	OR	p	95% CI
**Age**						
27-46	1.00	0.987	[0.4-2.6]			
47-66 (Ref)	1.00	-	-			
**Marital status**						
Single	2.22	0.101	[0.9-5.8]			
Married (Ref)	1.00	-	-			
**Child status**						
Have (Ref)				1.00	-	-
Don’t have				0.67	0.344	[0.2-1.0]
**Property ownership**						
Homeowner (Ref)	1.00	-	-	1.00	-	-
Renter	2.31	0.019	[1.1-4.7]	1.95	0.049	[1.0-3.8]
Work experience as a faculty member						
≤15 years	4.53	0.010	[1.4-14.4]			
≥16 years (Ref)	1.00	-	-			

Ref: Reference value

## Discussion

This study, carried out in the first-degree earthquake zone with a high level of risk at a university in Turkey, provides remarkable findings regarding the earthquake preparedness of faculty members. The study group would be expected to have a high level of earthquake preparedness because of their high education and income level. However, the current study showed that the faculty members did not implement many simple measures, so their earthquake preparations were insufficient.

According to the study, many faculty members stated that fulfilling earthquake preparedness measures is not challenging and that these measures are helpful. Approximately two-thirds of the faculty members said they read the materials about earthquake preparation or watched the media’s related news. About half of the study group received first aid training. These findings can be explained by the fact that the study group has the highest education level and more skills and opportunities to reach the information. It has been reported that formal education is an essential factor that positively affects disaster preparedness.^16^ However, although there were possibilities to reach these sorts of information, it had been shown that they were weak to take action. Their levels of preparedness were low, especially in terms of supplying necessary materials and fixation and planning parts of MLEPS. Only one faculty member (0.6%) had fulfilled all preparation criteria needed for surviving when an earthquake occurs. In a study conducted at a university in Turkey with 207 faculty members using the MLEPS scale, similar to the findings in the present study, the level of fulfilling the criteria for earthquake preparedness was deficient. In that study, the preparation level of the materials that can be used immediately after the earthquake was reported as 53% for flashlight, 46% for bottled water, 44% for first aid kit, 34% for canned food, 21% for a fire extinguisher. Also, the study was informed that less than 40% of faculty members fixed heavy objects in their homes.^22^ According to the study conducted by FEMA, 57% of the individuals prepared all the materials in their homes needed in case of a disaster; the percentages of the prepared materials by the individuals were 74% for canned food, 71% for bottled water, 42% for flashlight, 39% for first aid kit.^13^ In another study conducted with 1002 participants in Hong Kong, 57% of individuals prepared food and water, 49% of them had first aid kits, and 11.5% had fire extinguishers in their houses. The same study showed that approximately one-fourth of the individuals (26%) had first aid training.^23^ Moreover, in a study conducted with engineering students in Lebanon, serious inadequacies in earthquake preparation have been reported. In particular, 88% of the students felt insecure against a possible earthquake, 7% of them secured heavy furniture, 3% of them determined a safe place to shelter in the house during an earthquake, 15% of them prepared a flashlight with extra batteries, 11% of them had radio with extra batteries, 23% of them have a complete first-aid kit, 19% of them prepared bottled water and canned food has been reported.^24^

In this study, a renter increased the probability of low score by 2.31 and 1.95 times for materials preparedness and knowledge and skill, respectively. The percentage of the faculty members who had made their house controlled by the experts was 48.1% among the homeowners, while only 14.4% among the renters. In addition, the renters were less tending to fasten their tall furniture to the walls. Many studies have shown that property ownership affects the readiness of the disaster in positive ways.^15,25,26^ These findings indicate that renters were not as motivated as the homeowners in terms of taking responsibility; they preferred to avoid this kind of concern. However, there are some studies that earthquake preparedness is not related to property ownership.^18,19^

In this study, the work experience as a faculty member was another critical determinant of earthquake preparedness. Fifteen years or less of work experience increased the low material preparedness score’s probability of 4.53 times. This finding can be explained by further developing self-responsibility feelings, cognitive abilities, and information sources when the work experience increases.

This study, which was performed with a group of the highest level of education in a region with an increased risk of earthquakes in Turkey, shows that the faculty members’ level of fulfillment of the preparatory criteria for the earthquake was insufficient. Having a long working duration as a faculty member and homeownership affected the level of earthquake preparedness of them positively. Faculty members have an important position to increase the sensitivity of university students and the whole community regarding disaster preparedness. Hence, the motivation of faculty members to prepare for the earthquake should be strengthened. However, this study provides only the data obtained from the faculty members and does not give information about the level of earthquake preparedness of the general population. Therefore, to understand the capacity and motivation of earthquake preparedness of different social classes in Turkey and determine the best strategies for improving disaster preparedness, further studies are required.

**Acknowledgements**

We would like to thank Zerrin Yilmaz for her support in the data collection stage of the study. We also thank all of the faculty members for their participation in the study.
